# Molecular Detection and Sensitivity to Antibiotics and Bacteriocins of Pathogens Isolated from Bovine Mastitis in Family Dairy Herds of Central Mexico

**DOI:** 10.1155/2015/615153

**Published:** 2015-03-01

**Authors:** Ma. Fabiola León-Galván, José E. Barboza-Corona, A. Arianna Lechuga-Arana, Mauricio Valencia-Posadas, Daniel D. Aguayo, Carlos Cedillo-Pelaez, Erika A. Martínez-Ortega, Abner J. Gutierrez-Chavez

**Affiliations:** ^1^Food Department, Life Sciences Division, University of Guanajuato, Campus Irapuato-Salamanca, 36500 Irapuato, GTO, Mexico; ^2^Graduate Program in Biosciences, Life Sciences Division, University of Guanajuato, Campus Irapuato-Salamanca, 36500 Irapuato, GTO, Mexico; ^3^Agronomy Department, Life Sciences Division, University of Guanajuato, Campus Irapuato-Salamanca, 36500 Irapuato, GTO, Mexico; ^4^Department of Physics, University of Antwerp, Campus Groenenborger, Groenenborgerlaan 171, 2020 Antwerp, Belgium; ^5^Experimental Immunology Laboratory, National Institute of Pediatrics, Ministry of Health, 04530 México, DF, Mexico

## Abstract

Thirty-two farms (*n* = 535 cows) located in the state of Guanajuato, Mexico, were sampled. Pathogens from bovine subclinical mastitis (SCM) and clinical mastitis (CLM) were identified by 16S rDNA and the sensitivity to both antibiotics and bacteriocins of *Bacillus thuringiensis* was tested. Forty-six milk samples were selected for their positive California Mastitis Test (CMT) (≥3) and any abnormality in the udder or milk. The frequency of SCM and CLM was 39.1% and 9.3%, respectively. Averages for test day milk yield (MY), lactation number (LN), herd size (HS), and number of days in milk (DM) were 20.6 kg, 2.8 lactations, 16.7 animals, and 164.1 days, respectively. MY was dependent on dairy herd (DH), LN, HS, and DM (*P* < 0.01), and correlations between udder quarters from the CMT were around 0.49 (*P* < 0.01). Coagulase-negative staphylococci were mainly identified, as well as *Staphylococcus aureus*, *Streptococcus uberis*, *Brevibacterium stationis*, *B. conglomeratum*, and *Staphylococcus agnetis*. Bacterial isolates were resistant to penicillin, clindamycin, ampicillin, and cefotaxime. Bacteriocins synthesized by *Bacillus thuringiensis* inhibited the growth of multiantibiotic resistance bacteria such as *S. agnetis*, *S. equorum*, *Streptococcus uberis*, *Brevibacterium stationis*, and *Brachybacterium conglomeratum*, but they were not active against *S. sciuri*, a microorganism that showed an 84% resistance to antibiotics tested in this study.

## 1. Introduction

In Mexico, the national milk production has an average annual growth rate of ~1.3%, representing an increase of 9,784 to 10,677 million liters per year during the period from 2003 to 2010 [1]. The backyard livestock is one of the oldest production systems in Mexico; however, the governments have not considered it important enough [2]. In the last few years, family dairy herds or small-scale dairy enterprises contribute to the national milk production with values ranging from 35 to 40% [3]. Milk is mainly sold locally in different sale channels directly to consumers, or through intermediaries or the rural or commercial industry. Intermediaries collect milk either to supply fluid milk in urban areas or to manufacture traditional cheese that is in remarkable demand in cities or suburban areas [4, 5].

According to the Food and Agriculture Organization [6], small herds are a majority in the developing world. In these herds, animal health care is scarce because producers carry out neither preventive medicine nor a hygienic handling of milk during milking [4]. Even though mastitis is the largest cause of antimicrobial use in dairy herds [7, 8], very little is known about the use of antibiotics in small dairy herds. Mastitis is the inflammation of the mammary gland and it is a complex and costly disease in dairy herds [9, 10]. Subclinical mastitis (SCM) has a tendency to persist because it usually remains undetected. About 70 to 80% of the estimated $140 to $300 dollar loss per cow per year from mastitis relates to decreased milk production caused by asymptomatic subclinical mastitis [11]. The bacterial contamination of milk from the affected cows makes it unhealthy for human consumption and has zoonotic importance [12]. The mastitis occurrence in Mexico has been reported [13, 14], but there are few reports about bovine udder health, including the etiology of intramammary infections (IMI), antimicrobial susceptibility patterns, and mastitis frequency [15].

Alternatively, bacteriocins are antimicrobial peptides ribosomally synthesized by prokaryotes that inhibit or kill phylogenetically related and/or unrelated microorganism that share the same microbial niche. These peptides have a potential for diversified use in different areas such as food, pharmaceutical industries, agriculture, and apiculture [16, 17]. In particular, bacteriocins produced by* Bacillus thuringiensis, *the most important microbial insecticide, have showed potential to inhibit* Staphylococcus aureus* isolates associated with bovine mastitis [18]. Unfortunately, no other bacteria associated with this disease in Mexico have been tested using antimicrobial peptides synthesized by* B. thuringiensis*. In this study, our objective was to isolate and to identify molecularly microorganisms from bovine mastitis, determine antimicrobial susceptibility to antibiotic and bacteriocins synthesized by* B. thuringiensis*, and estimate the frequency of mastitis in family dairy herds from the central region of Mexico.

## 2. Material and Methods

### 2.1. Study Area and Herds

The study was developed in four municipalities in the state of Guanajuato, Mexico: Abasolo, Cuerámaro, Irapuato, and Silao. This region is located in central Mexico, to the south of the Mexican high plateau. Geographically, there are three climatic zones defined in Guanajuato with a pleasant climate with temperatures ranging from 11.7°C to 24.2°C, an average altitude of 2,015 meters above sea level, and annual average rainfall of 635 mm. Guanajuato is located at west longitude 99°40′–102°6′ and north latitude 21°51′–19°55′. Thirty-two family dairy herds were included in this study, which were selected for convenience based on the readiness to participate in the research and the existence of productive and reproductive data at the sampling time. All farms included in this study were classified as family dairy herds, according to [19], who report that farms, including the management system and facilities, should be directly served by the owner and family members, as is the case in the present study [20]. Most herds were Holstein-Friesian breed type with different herd sizes, cows with a different number of days in milk, number of calving, age, and level of milk yield.

### 2.2. Milk Sample Collection

Subclinical mastitis (SCM) was detected by reactive application (Masti test, BIVE, Mexico) to California Mastitis Test (CMT) in all lactation cows, including a total of 535 animals, following the method described by Schalm and Noorlander [21]. The results were interpreted in scores (range 0–4): 0 for no reaction, 1 a trace, 2 a weak positive, 3 a distinct positive, and 4 a strong positive, or in the case of clinical mastitis cases considering visual abnormalities such as flakes, clots, or any color changes in the milk, or by detecting slight swelling of the affected quarter udder. Once the udder quarters affected by subclinical (CMT 3) and clinical (any visual abnormality) mastitis were identified, teats were disinfected with swabs soaked in 70% ethyl alcohol. After discarding the first few streams, 10–15 mL milk samples were collected in sterile caped tubes and numbered, according to standard procedures of the National Mastitis Council [22]. Samples were cooled and immediately transported to the Laboratory of Proteomic and Genic Expression of the Life Science Division at the University of Guanajuato, Mexico.

### 2.3. Microbiological Culture and Isolation

Forty-six milk samples from udder quarters affected by mastitis were sent to microbiological analysis. Each sample was taken in clean conditions and a sown dilution of 1 : 10, 1 : 100, and 1 : 1000. The dilution was made using PBS buffer (130 mM NaCl_2_, 10 mM NaPO_4_, and pH 7.2) and then it was added to culture medium with agar as per standard procedures [22]. The different culture media used were Todd-Hewitt, Tryptic Soy Agar, and culture medium containing peptone trypticase, 10 g/L; yeast extract, 1.0 g/L; KH_2_PO_4_, 3.0 g/L; K_2_HPO_4_, 4.8 g/L; (NH_4_)_2_SO_4_, 30 g/L; MgSO_4_·7H_2_O, 0.2 g/L; L-cysteine HCl·H_2_0, 0.5 g/L; sodium propionate, 15 g/L; agar, 15 g/L; pH 6.0–7.9, all with the addition of 5% of sheep blood. The plates were incubated under aerobic conditions at 37°C for 72 h. For molecular identification those culture plates with growing of one or two different colonies were included. Culture plates showing the growth of three or more different colonies were discarded and registered as contaminate sample [22]. They also were subcultured in LB liquid at 37°C for 72 h, and after this time 20% glycerol was added. Bacteria stocks were stored at −80°C.

### 2.4. 16S rDNA Amplification

For confirmation of the identity, isolation of genomic DNA was carried out by picking one colony from fresh culture plate. The 16S rDNA was amplified by colony-PCR using 10 pM of the universal oligonucleotide set that amplifies both bacterial domains: forward UBF 5′-AGAGTTTGATCCTGGCTGAG-3′ and reverse 1492 R5′-GGTTACCTTGTTACGACTT-3′. For the amplification of 16S rDNA a proof fidelity enzyme (BioRad) was used under the following conditions: 5 min at 95°C; 30 cycles of 30 s at 95°C, 30 s at 58°C, and 1 : 30 min at 72°C; and finally 5 min at 72°C. An aliquot of 5 *μ*L of the PCR products was subjected to electrophoresis in 1% agarose gels and stained with ethidium bromide to visualize the amplified products. The sequencing was performed in Molecular Cloning Laboratories (MCLAB; San Francisco, CA, USA). Amplicons were treated for analysis restriction of amplified fragments (ARDRA) with 10 U of enzymes* Mbo*I and* Bam*HI (New England, Bio-Lab UK). The digestion reaction was performed at 37°C for 3 h. The digestion products were analyzed in 2% agarose gels stained with ethidium bromide and digitalized. The amplified fragment from microorganisms that presented different restriction patterns was selected for sequencing. The sequencing was performed in Molecular Cloning Laboratories (MCLAB; San Francisco, CA, USA).

### 2.5. Bioinformatics Analysis

The ambiguous bases from the 5′ and 3′ terminal sequences were eliminated, and the resulting sequences were confirmed using BioEdit software. Sequences were then compared against the Ribosomal Database Project and GenBank using BLAST against the NCBI nonredundant nucleotide database “nt.”

### 2.6. Antibiotic Susceptibility Testing

For susceptibility testing, isolates were suspended in 5 mL trypticase soy broth (TSB) at 28°C or 37°C to a turbidity of 0.5 on a scale of McFarland and with a sterile swab extension covered by the surface of a Petri dish with Muller-Hinton agar gel (MH) (Difco). The antibiotic susceptibility was identified by routine diagnostic methods using standard disk diffusion for Gram-positive and Gram-negative (MultiBac I.D., México D.F). Zones of inhibition (in mm) were recorder after ~18 h of incubation at 35–37°C. The zones of inhibition (mm) were determined and compared with the standards of performance of the supplier to determine whether the tested strain was sensitive (S), intermediate (I), or resistant (R).

### 2.7. Susceptibility to Antimicrobial Peptides of* B. thuringiensis*

Mexican strains of* B. thuringiensis* subsp.* morrisoni*,* B. thuringiensis* subsp.* kurstaki*,* B. thuringiensis* subsp.* kenyae*,* B. thuringiensis* subsp.* entomocidus*, and* B. thuringiensis* subsp.* tolworthi* produce the bacteriocins Morricin 269, Kurstacin 287, Kenyacin 404, Entomocin 420, and Tolworthcin 524, respectively. These bacteria were cultured at 28°C, 200 rpm, for 24 h in tryptic soy broth (TSB). Extracellular proteins were precipitated with ammonium sulfate to 80% saturation at 4°C, resuspended in 100 mM phosphate buffer (pH 7.0), and dialyzed overnight using a 1 kDa cut-off membrane (Amersham Biosciences) to obtain partially purified bacteriocins. To carry out the well-diffusion assay, indicator bacteria were cultivated overnight in tryptic soy broth (TSB), and 105 *μ*L (~1 × 10^9^ cell/mL) of each culture was mixed with 15 mL of TSB with warm soft agar 0.7% (w/v) and plated. Five wells of 8 mm in diameter were dug into the agar and 100 *μ*L of partially purified Morricin (~150 U), Kenyacin (~260 U), Entomocin (~260 U), Tolworthcin (~260 U), and Kurstacin (~360 U), whose inhibitory activities were standardized with* Bacillus cereus* 183 as indicator bacterium, was added to each well. Then samples were incubated for 12 h at 4°C to allow diffusion of samples, followed by an additional incubation at 28°C or 37°C for 1 day before diameters of zones of inhibition were measured. The minimum detectable zone measured for analytic purposes was 1 mm beyond the well diameter. One unit (U) of bacteriocin activity was defined as equal to 1 mm^2^ of the zone of inhibition of growth of the target indicator bacterium [17, 18, 21]. Additionally, the inhibitory effect of bacteriocins against bacteria was also performed using gel-screening assay. Partial purified bacteriocins in Laemmli's buffer without *β*-mercaptoethanol were loaded in two continuous sodium dodecyl sulfate- (SDS-) polyacrylamide gels for electrophoresis (SDS-PAGE). One gel was stained with Coomassie blue and the other was fixed in 25% (v/v) isopropanol and 10% (v/v) acetic acid. The gel was washed with double-distilled water and equilibrated in phosphate buffer (pH 6.5). The gel was overlaid with TSB with soft agar 0.7% (w/v) containing ~1 × 10^9^ cell/mL of indicator bacteria and incubated at 28°C. The next day zones of inhibition were examined and molecular mass of the bacteriocins was calculated [17].

### 2.8. Data and Statistical Analyses

Data registered in the herds were entered into a spreadsheet in electronic format with Excel for Windows and edited to guarantee the quality of analyses. The dependent variables studied were the test day milk yield in kg (MY) and CMT results by udder quarter. Independent variables were lactation number (LN), family dairy herd (DH), herd size (HS), number of days in milk (DM), and municipality (M). Descriptive analysis was used for the variables included in this study. Normality was evaluated for the dependent variables to define the type of statistical analysis. The variables were recorded and grouped into the next categories: HS (0–15, 16–25, and >25 cows), LN (1, 2, 3, and >4), and DM (0–90, 91–180, and >180 days). In order to know the independence between some variables and because most of these were discrete and without normal distribution, the chi-square test was applied between MY, DH, HS, LN, and DM. To estimate the probability of association between results of CMT, udder quarters were estimated using the Spearman rank correlation. For the statistical analyses, the Statgraphics Centurion program version 15.2 was used.

## 3. Results and Discussion

### 3.1. Characteristics and Parameters of Dairy Farms

Table 1 shows the variability among farms according to the herd size (from 3 to 47 heads), number of lactations (from 1.7 to 4.1 lactations), number of days in milk (from 52 to 275 days), and average of test day milk yield (from 9.0 to 26.4 Kg). The family dairy herds are one of the dominant and widely distributed production systems in Mexico, in small scale units run by the family. It was estimated that 10% of all milk production in Mexico comes from family dairy herds. According to the livestock census carried out in 2007, it was found that ~73% of units correspond to the small farms [4]. It is interesting that although in Mexico a decrement in the family dairy participation has been reported to domestic supplies it has been observed that it does not have a direct influence on the number of small farms as it remains without important changes. The herd size of the family farms reported in this study is much lower than suggested from family dairy farms in Los Altos, Jalisco, Mexico, where an average population of 61 lactating cows was described. However, the average of milk yield per cow obtained in this study (20.6 L/d) was higher than 17.8 L/d (Jalisco) [23] and 11.4 L/d (Jalisco and Michoacán) [24]. As indicated above, of all the dairy production systems in Mexico, the familial system is the most heterogeneous. The farms that compose this system range from subsistence operations (milk and cheese are used exclusively to feed the family) to large-scale operations (milk sale is the primary but not the unique source of income for the family). The family production system is centered in the west central region of the country, including the states of Jalisco, Michoacán, Aguascalientes, and Guanajuato [23].

### 3.2. Frequencies of Subclinical and Clinical Bovine Mastitis

CMT global results in this study showed that 48% of animals (*n* = 257) were negative, and 52% of animals (*n* = 278) showed a positive reaction to SCM. However, SCM per animal among herds ranged from 11 to 75%. Concerning the quarter reaction degree of CMT, milk samples registered a 10.3% (trace), 6.12% (grade 1), 2.9% (grade 2), 5.28% (grade 3), and 1.59% dry-off gland, and the remaining percentage was negative (72.2%). In general, at least one case of clinical mastitis was detected in 66% of the dairy herds studied (21 of 32). The percentage of CLM per animal among herds ranged from 0 to 25 (Table 2). In small-scale dairy herds, hygiene and health management are often poor, a situation that contributes to the development of clinical mastitis cases [13]. This might explain the higher prevalence of CLM registered in this study (0–25%), especially when datum is compared with the results obtained in family dairy herds from State of Mexico (3.4–9.8%) [13] and also with Jalisco (4.0%) [25]. Furthermore, it is necessary to consider that there is a clear variation in the epidemiology of mastitis and mastitis inducers among different regions in Mexico [25]. The frequency of clinical cases based on quarter udder signs was 78% (25/32), moderately acute; 16% (5/32), chronic; and 6.2% (2/32), severe acute. Statistical analysis showed that MY was dependent on the farm, HS, DIM, and LN (*P* < 0.01) (Table 3). The estimated correlation between results of CMT for each udder quarter ranged from 0.46 to 0.52 (*P* < 0.01). Mastitis is an expensive disease, where a high proportion of dairy farms might have avoidable losses [26]. The frequency of SCM obtained in our study (11–75%) was as high as that obtained (23–52%) in dairy cattle located in province of Huaral, Lima, Peru [27]. In addition, our data are comparable with two studies carried out in smallholder and/or family dairy farms located in the Jalisco and State of Mexico, Mexico, where SCM prevalence per animal was of 34.1 and 48.3%, respectively [13, 25]. It is important to indicate that both SCM and CLM were associated with herd size, parity, management practices, and time of lactation [25]. The prevalence of mastitis might change between countries and geographical regions, but frequently the highest prevalence is found in countries with a poorly developed dairy sector and with a lack of udder health control programs.

### 3.3. Isolation and Bacterial Identification

A total of eleven milk samples plated (24%) were selected for bacterial isolation and identification. The remaining milk samples plated were not considered for showing a lack of growth or a contaminated bacteria growth. It is necessary to highlight that most of the milk samples of this study were from SCM cases, where (i) the colony-forming units of the organism in the milk were below the detection limit of the assay, (ii) special media or growth conditions were required, or (iii) presence of inhibitors in the milk sample, such as antibiotics, had interfered with the growth of the pathogen. If it is common that 20–30% of clinical quarters will result in no microbial growth, this percentage could be increased when milk samples come from SCM as in this study. Also, clinical signs could be present but the pathogen might be eliminated or controlled by the cow's immune system [22]. Bacterial PCR amplification and subsequent ARDRA analyses of 16S rDNA gene were successful for all samples. The 16S rDNA sequences that presented different ARDRA profiles were selected for sequencing (Table 4). Five genera and eleven bacterial species involved in cases of mastitis were identified. Table 4 shows that 42% of the isolated microorganisms were coagulase-negative staphylococci (CNS). Similar results were obtained in smallholder dairy farms from Jalisco state of Mexico, where the most common udder pathogens were CNS (15.6%), followed by* S. aureus* (5.9%),* S. agalactiae *(6.8%),* Corynebacterium* spp. (14%), and coliform bacteria (4.1%) [25]. CNS are considered minor pathogens, especially in comparison with major pathogens such as* S. aureus*, streptococci, and coliforms [28]. However, these bacteria are of great interest because they are regularly isolated from milk samples obtained from cows and are currently considered emerging pathogens of bovine mastitis and the main cause of intramammary infection (IMI) in modern dairy herds [29–31].

Alternatively, a total of 124 milk samples were collected from 124 multiparous lactating dairy (Holstein) cows at the province of Nanning, China. Positive CMT was recorded from 65 (52.4%) glands. Bacteria were isolated from 45 (36.3%) of milk samples. Distributions of microbial isolates responsible for infected milk samples have been reported as follows:* S. aureus* (47%), CNS (27%),* Escherichia coli* (9%),* S. agalactiae* (9%),* S. uberis* (4%), and* Cryptococcus neoformans* (4%) [32]. In another study, Lago et al. [33] found 422 cows affected by clinical mastitis in 449 quarters, where coliform bacteria were the most commonly isolated pathogen (24% of clinical mastitis cases).

According to results of sequence analysis of isolates conducted in the present study (retrieved from the GenBank, http://www.ncbi.nlm.nih.gov/, using the nucleotide-nucleotide BLAST algorithm)* Staphylococcus agnetis *(NCBI/EMBL accession JQ 394696) was isolated from milk samples of mastitis cases. It is important to emphasize that* S. agnetis* is mentioned only once before in the literature as a pathogen causing mastitis in dairy cattle. Recently, it has been reported that* S. agnetis* was associated with bovine mastitis based on the characteristics of 12 isolates originating from milk samples of cows with subclinical or mild clinical IMI and one isolate from the apex of the teat [34]. We also identified the bacteria* Brevibacterium stationis* and* Brachybacterium conglomeratum*. Although these microorganisms have not been reported as etiological agents of cow mastitis, they have occasionally been isolated from goat raw milk samples and also from different areas of the farm (e.g., teat surfaces, milking parlors, hay, air, and dust) [35].

### 3.4. Antibiotic Susceptibility Patterns

**Figure 1:**
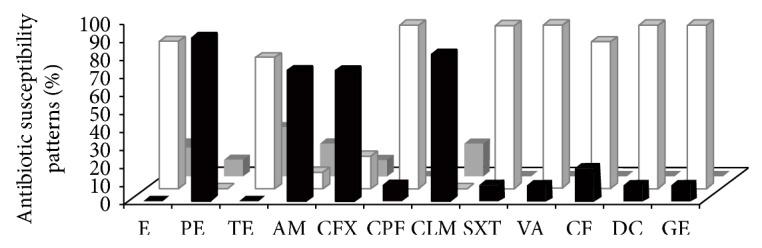
Percentage of sensitivity* in vitro* by standard disk diffusion (MultiBac-ID) of different antibiotics against bacterial isolates from bovine mastitis. Graphic bars represent the percentage of sensitive (white), intermediate (grey), or resistant (black). Erythromycin (E), penicillin (PE), tetracycline (TE), ampicillin (AM), cefotaxime (CFX), ciprofloxacin (CPF), clindamycin (CLM), sulfamethoxazole-trimethoprim (SXT), vancomycin (VA), cephalothin (CF), dicloxacillin (DC), and gentamicin (GE).

Six isolates of this study (54.54%) showed resistance to two or three antimicrobial agents, mostly to penicillin, clindamycin, and cefotaxime; meanwhile resistance to four or more antimicrobial agents was found in 5 isolates (45.45%). All isolates showed a variable susceptibility (~60%) to the 12 antimicrobials tested. Special consideration showed* Staphylococcus sciuri* isolated that was resistant to 10 of 12 antimicrobials tested and the rest were detected with intermediate susceptibility (Table 4). The microorganisms were mainly resistant to penicillin (90%), clindamycin and cefotaxime (both 80%), and ampicillin (70%). In this study we found a high frequency of penicillin-resistant bacteria, which is higher than those reported in subclinical milk samples obtained from dairy herds located in state of Michoacán, Mexico (74%) [18]. In addition, percentage of ampicillin-resistant microorganisms (i.e., 70%) was very similar to that reported (67.4%) in a study performed in dairy herds of south of Brazil [36]. In particular, in Mexico it is very common that, at the end of period of lactation in dairy cattle, farmers use a prophylactic dose of antimicrobial (i.e., penicillins and cephalosporins) into the udder. Although the purpose of this treatment is the prevention of future mastitis, it is obvious that this procedure might generate penicillin-resistant microorganisms [37]. In addition, some isolates were highly sensitive (90%) to trimethoprim/sulfamethoxazole, dicloxacillin, ciprofloxacin, and gentamicin (Figure 1). All microorganisms shown in Table 4 were identified as Gram-positive bacteria. Only one Gram-negative microorganism was isolated and identified as* Raoultella* sp., which had resistance to ampicillin, carbenicillin, and cephalothin.* Raoultella* sp. (before* Klebsiella* sp.) is one of the most frequent Gram-negative pathogens isolated from bovine clinical mastitis [38]; and it has been isolated from bedding material [39].

The emergence of antimicrobial resistance among pathogens that affect animal health is a growing concern in veterinary medicine. Furthermore, the use of antimicrobial drugs has also been considered as a potential health risk for humans [40, 41].* S. agnetis* showed resistance (33.0%) to penicillin, ampicillin, cefotaxime, and clindamycin (Table 4). It should be noted that, in another study,* S. agnetis* was resistant to lysozyme, polymyxins, and deferoxamine, and it was susceptible to novobiocin and lysostaphin [34]. Phylogenetically,* S. agnetis* is a novel species of the genus* Staphylococci* and can be differentiated from the coagulase-positive species, such as* S. hyicus, S. simulans, S. schleiferi, S. chromogenes, S. intermedius,* and* S. epidermidis*. Compared to* S. aureus*, streptococci, and coliforms, coagulase-negative staphylococcus (CNS) has been considered an emerging bovine mastitis pathogen in several countries [30, 42, 43] with a high degree of resistance to some conventional drugs [30, 40, 43, 44]. CNS mastitis responds much better to antimicrobial treatment than* S. aureus* mastitis, but resistance to different antimicrobials is more common in CNS than* S. aureus*. CNS tends to be more resistant to antimicrobials than* S. aureus* and can easily develop multiresistance. The most common resistance mechanism in staphylococci is *β*-lactamase production, which results in resistance to penicillin G and aminopenicillin [28].

### 3.5. Inhibitory Activity of Bacteriocins

We recently showed that antimicrobial peptides or bacteriocins (Morricin 269, Kurstacin 287, Kenyacin 404, Entomocin 420, and Tolworthcin 524) synthesized by* B. thuringiensis *are able to inhibit food-borne pathogenic bacteria [17]. In addition it was demonstrated that* Staphylococcus* strains isolated from bovine with mastitis are also susceptible to this kind of bacteriocins [18]. In the present study the five bacteriocins inhibited the growth of* S. agnetis, S. equorum, Streptococcus uberis, B. stationis*, and* B. conglomeratum*, bacteria that showed multiantibiotic resistance (Table 5). Unfortunately, bacteriocins did not show activity on* S. sciuri, *microorganism with an 84% resistance to antibiotics tested in this study. This bacterium has been found to be associated not only with bovine subclinical mastitis [45], but also with serious infections in humans such as endocarditis [46], peritonitis [47], wound infections [48], and urinary infections [49]. In addition, we did not find susceptibility of* S. aureus *to the bacteriocins, which is very interesting as we previously demonstrate that different isolates of this bacterium are susceptible to the five antimicrobial peptides tested in this work [18]. We do not have a clear explanation for this observation, but it has been shown that, within the same genus or strains of the same species, microorganisms can differ in their susceptibilities to a particular bacteriocin. For example, (i)* B. licheniformis* strain P40 produces an antimicrobial peptide with inhibitory action to* S. intermedius* but not to* S. aureus* [50]. (ii) Also,* S. aureus *strains isolated from dairy cow mastitis [18] showed different susceptibilities to the five bacteriocins used in this study. In addition, it seems that pathogenic microorganisms have acquired the ability to sense and to respond to bacteriocins in different way, often resulting in reduced negative charge of their cell envelope due to specific surface modifications, which in consequence induce the generation of bacteriocin-resistant bacteria [51].

**Figure 2:**
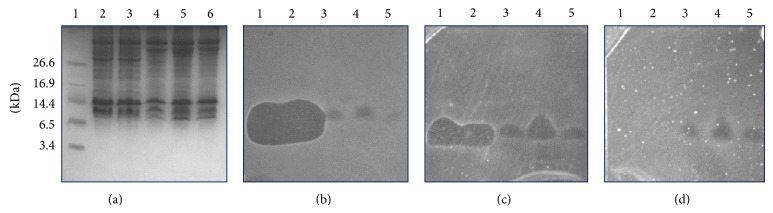
Inhibitory detection of bacteriocins against bacteria using gel-screening assay. (a) SDS-PAGE; gel was overlaid with (b)* Bacillus cereus* 183 (control), (c)* Raoultella* sp., and (d)* Staphylococcus agnetis*. Bacteria (c) and (d) were isolated from bovines with mastitis. Lane 1, Morricin 269; lane 2, Kurstacin 287; lane 3, Kenyacin 404; lane 4, Entomocin 420; lane 5, Tolworthcin 524. Growth inhibition zones show the relative position of bacteriocins with molecular mass of ~10 kDa. Protein marker (BioRad) was used to estimate the molecular masses of bacteriocins.

Alternatively, in order to detect the molecular mass of the bacteriocins, we carried out gel-screening assays using* Raoultella *sp. and* S. agnetis *as reporter bacteria. Morricin 269, Kurstacin 287, Kenyacin 404, Entomocin 420, and Tolworthcin 524 exhibited molecular mass of ~10 kDa as shown previously (Figure 2) [17]. It is important to indicate that because we used different units of bacteriocins, we did not carry out comparisons in the inhibitory effects of the different bacteriocins against the bacteria assayed in this work, as our purpose was only to detect whether microorganisms were susceptible or not to the antimicrobial peptides.

## 4. Conclusions

In this work, the most common udder pathogens isolated from mastitis milk samples were coagulase-negative staphylococci (42%), followed by streptococci (17%), and* S. aureus, B. stationis, B. conglomeratum*, and* Raoultella* sp. with an 8% each. We found that 72.7% of isolates had a resistance pattern to three or more antimicrobial agents mainly to penicillin, clindamycin, and cefotaxime. Studies on the prevalence rate of clinical and subclinical mastitis of different mastitis pathogens in a cow population from small-scale dairy herds are scarce. Although it is difficult to compare results obtained in this work with those obtained in other countries, CNS,* S. aureus*, and streptococci have been reported to be the most prevalent pathogens [52, 53]. Alternatively, bacteriocins of* B. thuringiensis *inhibited the growth of different bacteria tested here and they could have a viable potential for use in integrated management programs to control or prevent mastitis in animals. However, it is obvious that a higher number of bacterial isolates with different genus or different strains of the same genera and species obtained from bovine mastitis must be tested in future studies.

## Figures and Tables

**Table 1:** Descriptive statistics for variables studied from family dairy herds (*n* = 32) from the central region of Mexico.

Parameter	Mean	Standard deviation	Minimum	Maximum
Herd size (heads)	16.7	9.4	3	47
Days in milk	161.4	108.2	7	730
Lactation number	2.8	1.6	1	16
Milk yield (Kg)	20.6	7.2	3	45

**Table 2:** Frequency of subclinical (SCM) and clinical (CLM) mastitis in family dairy herds from the central region of Mexico.

Frequency (%)^*^		
Farm	SCM	CLM
1	4/12 (33)	1/12 (8)
2	0/3 (0)	0/3 (0)
3	4/8 (50)	2/8 (25)
4	8/14 (57)	2/14 (14)
5	4/9 (44)	0/9 (0)
6	5/9 (56)	0/9 (0)
7	3/14 (21)	0/14 (0)
8	5/17 (29)	0/17 (0)
9	10/16 (63)	1/16 (6)
10	5/10 (50)	0/10 (0)
11	9/26 (35)	2/26 (8)
12	6/16 (38)	0/16 (0)
13	3/11 (27)	0/11 (0)
14	9/12 (75)	3/12 (25)
15	13/28 (46)	6/28 (21)
16	7/13 (54)	2/13 (15)
17	19/31 (61)	6/31 (19)
18	2/12 (17)	2/12 (17)
19	3/19 (16)	1/19 (5)
20	8/20 (40)	3/20 (15)
21	8/47 (17)	9/47 (19)
22	17/36 (47)	4/36 (11)
23	2/18 (11)	4/18 (22)
24	18/29 (62)	0/29 (0)
25	3/12 (25)	1/12 (8)
26	7/15 (47)	2/15 (13)
27	5/9 (56)	0/9 (0)
28	10/16 (63)	2/16 (13)
29	3/9 (33)	1/9 (11)
30	4/24 (17)	4/24 (17)
31	3/6 (50)	0/6 (0)
32	2/14 (14)	1/14 (7)

^*^Denominator represents the total number of animals in the herd.

**Table 3:** Results of the chi-square independence test between different variables.

Variables	Statistical values	Probability
Milk yield—herd	1684.197	0.0000
Milk yield—herd size	1706.651	0.0064
Milk yield—days in milk	2548.930	0.0000
Milk yield—lactation number	533.235	0.0000

**Table 4:** Potential microbial pathogens isolated from dairy cattle and their susceptibility to antibiotics^a^.

Bacteria	Accession number	Antibiotics^b^											
Gram-positive		E	PE	TE	AM	CFX	CPF	CLM	SXT	VA	CF	DC	GE

*Staphylococcus aureus *	KP224443	S	R	S	R	R	S	R	S	S	S	S	S
*Staphylococcus agnetis *	JQ394696	S	R	S	R	R	S	R	S	S	S	S	S
*Staphylococcus epidermidis *	KP224442	S	R	S	I	R	S	R	S	S	S	S	S
*Staphylococcus sciuri *	KP224448	I	R	I	R	R	R	R	R	R	R	R	R
*Staphylococcus haemolyticus *	KP224444	S	R	S	R	R	S	R	S	S	S	S	S
*Staphylococcus equorum *	KP224447	S	R	S	R	S	S	I	S	S	S	S	S
*Streptococcus dysgalactiae *	KP224445	S	R	S	R	R	S	R	S	S	S	S	S
*Streptococcus uberis *	KP224446	S	I	S	R	S	S	I	S	S	R	S	S
*Brevibacterium stationis *	KP224449	I	R	S	I	I	S	R	S	S	S	S	S
*Brachybacterium conglomeratum (1)*°* cvbnm *	KP224450	S	R	I	S	R	S	R	S	S	S	S	S

Gram-negative		CL	AK	CB	NET	NF	NOF	CF	AM	CFX	CPF	SXT	GE

*Raoultella *sp.	KP224451	S	S	R	S	I	S	R	R	S	S	S	S

^a^R, resistant; S, susceptible; I, intermediate.

^b^Erythromycin (E), penicillin (PE), tetracycline (TE), ampicillin (AM), cefotaxime (CFX), ciprofloxacin (CPF), clindamycin (CLM), sulfamethoxazole-trimethoprim (SXT), vancomycin (VA), cephalothin (CF), dicloxacillin (DC), gentamicin (GE), amikacin (AK), carbenicillin (CB), chloramphenicol (CL), netilmicin (NET), nitrofurantoin (NF), and norfloxacin (NOF).

**Table 5:** Inhibitory activity (U^a^) of partial purified bacteriocin determined by the well-diffusion method against potential microbial pathogens associated with mastitis in dairy bovines.

Indicator bacteria	Bacteriocins				
	Morricin 269	Kurstacin 287	Kenyacin 404	Entomocin 420	Tolworthcin 524
*Bacillus cereus 183* ^ b^	151	365	264	264	264
*Staphylococcus aureus *	0	0	0	0	0
*Streptococcus dysgalactiae *	0	0	365	365	330
*Staphylococcus agnetis *	53	28	142	148	104
*Staphylococcus epidermidis *	0	0	0	0	0
*Streptococcus uberis *	204	296	264	296	233
*Staphylococcus sciuri *	0	0	0	0	0
*Staphylococcus haemolyticus *	0	0	0	0	0
*Staphylococcus equorum *	186	245	231	374	225
*Brevibacterium stationis *	62	28	329	204	150
*Brachybacterium conglomeratum *	103	44	296	480	150
*Raoultella *sp.	264	264	296	296	264

^a^One unit is defined as 1 mm^2^of the zone of inhibition as determined by the well-diffusion method (see text). Data are the average of triplicate assays. A value of “0” indicates no inhibition.

^b^Bacterium used as positive control. It was used to determine the units of bacteriocins contained in the crude extracts used in the assay [17, 18].
